# Two Cases of Hydrophobia Recently Observed in Smyrna

**Published:** 1855-08

**Authors:** 


*Two Cases of Hydrophobia recently observed in Smyrna.*—In the month of September, 1853, a dog bit a man, 22 years of age, on a naked part of the calf of the leg, and on the same day bit a female, 20 years of age, on the hand. The man's wound was deep. No caustic was applied. It suppurated freely, and did not cicatrize for fifteen days. The girl's wound was so slight that it was scarcely perceptible six or eight hours afterwards, when it was seen by a medical man, who touched it with nitrate of silver very slightly. The next day a young man was bitten in the hand, but the wound was immediately cauterized by the acid nitrate of mercury. The dog, being ill, was poisoned by strychnia. In April, 1854, eight months after the bite, the man first bitten was seized with unequivocal symptoms of hydrophobia, and died 70 hours after the symptoms declared themselves. On the 17th of April, 1855, twenty months after the bite, the girl also was attacked, and died in about 60 hours. The third person bitten has not yet suffered. The girl had not been aware that her bite might prove dangerous, and had had no fear of hydrophobia. Pustules under the tongue were looked for in the girl's case, but not found, although the arteries and veins of the part appeared congested. No unusual mode of treatment was practised. Dr. Macraith shortly alluded to three other cases he had heard of, showing that the disease was not unfrequent in Smyrna, and stated that a part of the coast of Asia Minor, opposite Rhodes, where hydrophobia was present in the days of Hippocrates, was still noted for the frequency with which it is met with in the present day.

Mr. Spencer Wells, having inquired of Dr. Macraith whether the symptoms in these cases had been preceded by pain in the bitten part, and received the reply that they had, in both instances, alluded to the propriety of excising the cicatrix, at any period after the bite, even after the commencement of the symptoms of hydrophobia He said pain in the cicatrix was so commonly observed, that, although it was difficult to suppose that a poison could be mechanically imprisoned in any part of the body, it appeared advisable to perform excision, if only for the purpose of giving confidence to the patient. In the only case of hydrophobia he had ever seen this was done, and the symptoms certainly abated for a few hours afterwards.

Mr. Eddowes said that in tetanus he would also perform excision of any injured part, and had seen the practice of great use, accompanied by the endermic application of morphia.

Mr. Halke alluded to the state of the kidneys in hydrophobia. He had seen a case last year in King's College Hospital, in which great diminution of urine, and finally suppression, took place. Urine drawn off by the catheter was smoky, highly albuminous, and largely charged with free epithelium and epithelial casts. The kidneys, at the post-mortem examination, presented all the appearances of acute desquamative nephritis. In another case, at Charing Cross Hospital, the kidneys presented similar appearances.

Dr. Leared suggested that possibly the diminution in the quantity of urine might depend upon the fact that no water was imbibed.

The Chairman inquired if any account could be given of the manner in which the dog acquired the disease. Dr. Macraith replied that it was a sporting dog, and nothing could be discovered as to inoculation.

Mr. McDonnell related a case he had seen to prove that the bite of a dog simply enraged, and not suffering from hydrophobia, might give rise to the disease. In this case the bite of a spaniel that continued well was followed by fatal hydrophobia.

Mr. Macleod said that this was a well known and established fact.

Mr. Spencer Wells doubted the correctness of these statements, and thought that a dog must be suffering from this specific disease before he could convey it to the human subject. He might have it mildly, (just as a person with but a few spots of small-pox might communicate a severe form of disease to another person), and recover. In the case he had seen, inoculation was distinctly traceable. It occurred in Malta, where the disease had been unknown for upwards of thirty years, and it was only after considerable trouble that it was found that a dog had arrived in a vessel from Gibraltar that had died of hydrophobia, after biting another dog, that had bitten a cat, whose bite had proved fatal to a woman after an interval of about six weeks.—*Ibid.*

